# Association between Sociodemographic Factors and Dietary Patterns in Children Under 24 Months of Age: A Systematic Review

**DOI:** 10.3390/nu11092006

**Published:** 2019-08-26

**Authors:** Gutiérrez-Camacho Claudia, Méndez-Sánchez Lucia, Klünder-Klünder Miguel, Clark Patricia, Denova-Gutiérrez Edgar

**Affiliations:** 1 Physical Therapy Research Unit, Hospital Infantil de México Federico Gómez, and Medicine Faculty of Autonomous National University, Mexico City 06720, Mexico; 2 Clinical Epidemiology Research Unit, Hospital Infantil de México Federico Gómez, and Medicine Faculty of Autonomous National University, Mexico City 06720, Mexico; 3 Research Headmaster’s Office, Hospital Infantil de México Federico Gómez, Ciudad de México 06720, Mexico; 4 Nutrition and Health Research Center, National Institute of Public Health, Cuernavaca 62100, Morelos

**Keywords:** dietary patterns, systematic review, children under 24 months old, sociodemographic factors

## Abstract

Background: Understanding early-life complementary feeding dietary patterns and their determining factors could lead to better ways of improving nutrition in early childhood. The purpose of this review was to evaluate evidence of the association between sociodemographic factors and dietary patterns (DPs) in children under 24 months. Methods: Medline (PubMed), Cochrane Central, NICE guidelines, and Trip database were searched for observational studies that evaluated sociodemographic factors and their associations with DP. Results: Seven studies were selected for the present review. High education level among mothers was inversely associated with unhealthy DPs and positively associated with healthy DPs. Higher household income was negatively associated with unhealthy DPs. Four studies showed a positive association between low household income and unhealthy DPs and three studies showed a positive association between higher household income and healthy DPs. Additionally, in younger mothers, body mass index (BMI ≥ 30.0 kg/m^2^) and number of children were positively associated with unhealthy DPs. Conclusions: This review provides evidence of a positive association between mothers’ higher education level, higher household income, higher maternal age, and healthy dietary patterns as well as a negative association between these factors and unhealthy dietary patterns. Further studies from low- and middle-income countries are needed for comparison with associations showed in this review.

## 1. Introduction

Good nutrition through childhood, particularly during the first two years, is crucial to optimum growth, health, and behavioral development [1]. In this sense, during the first six months of life, breast milk fulfills infants’ energy and nutrition needs. After six months, complementary feeding, defined as the inclusion of foods and liquids along with breast milk, must guarantee growth and development through the child´s remaining first two years [1,2,3]. Unfortunately, failure to meet these requirements through complementary feeding can often cause nutrient deficiencies, suboptimal growth, delayed development and disease, and may be due to a lack of variety and frequency in the types of foods provided to young children [4,5,6,7]. Sometimes infants cannot be feeding with maternal milk due to maternal human immunodeficiency virus infection (HIV), maternal death or non-intention to breastfeed, thus formula milk would be considered in addition to complementary diet [2].

In general, complementary feeding has been assessed in terms of variety of foods and nutrient needs. For many years, this representation has been considered an important health indicator where each nutrient has a function in the health–disease process [8,9,10,11,12,13]. However, more recently, dietary pattern (DP) analysis, conceptually defined as representations of nutrient and food consumption groups, has emerged as an option to predict risk of disease better than individual foods or nutrients. Therefore, understanding early-life complementary feeding DPs, their determining factors, and their influence on later health could lead to better ways of improving nutrition in early childhood [14,15,16]. Since DPs are difficult to measure, different methodologies (“a priori” and “a posteriori”) have been used to derive DPs. For the “a posteriori” approaches, multiple statistical techniques such as factor analysis, cluster analysis, and reduced rank regression analysis [17,18,19,20] have been employed to characterize DPs.

On the other hand, many complex social and demographic factors have been linked to the overall health of a population. The broad concept of socioeconomic status (SES) encompasses some of these factors, including parental education level, occupation, and family income, among others [21]. For this reason, SES should be understood as a compound measure of an individual’s economic and sociological status that has great impact on the health of the population; however, we have to consider the influence of many other factors associated with DPs in early childhood. Previous studies have reported evidence of diet-socioeconomic status gradients across all levels of human development and that lower socioeconomic status is strongly linked to unhealthy DPs [22]. These studies have also described unhealthy DPs in children from low-income and poorly educated families [23]. Nonetheless, studies in low- and middle-income countries have contradicted the correlation between socioeconomic factors (income and education) and DP [24,25].

Additionally, maternal and family influences on complementary feeding practices are considerably more important than other factors. Women who follow healthy dietary recommendations are more likely to have infants whose diets are compatible with “infant guideline” pattern scores [26]. On the other hand, women whose diets are characterized by unhealthy food consumption are more likely to have infants with similar DPs, some of which are detectable even before birth [27,28].

In this context, links have been reported between younger mothers (<30 years old), multiparity, smoking during pregnancy, body mass index (BMI) ≥30.0 kg/m^2^), and unhealthy DPs [29,30,31,32]. In general, “healthy diets” are found to go hand in hand with higher household income while “unhealthy diets” have been linked to lower household income [25,33]. The literature suggests that in families with low household income, children’s diet is characterized by low consumption of fruit and vegetables and high intakes of ultra-processed foods (commonly energy-dense foods), which have been linked to increased risk of children becoming overweight or obese [32,34]. Other sociodemographic factors, such as place of residence (rural or urban), number of children in the family, employment status, and parents’ diet have been suggested as important elements to complementary DP composition in children [35,36,37,38]. In addition, healthy DPs high in fruit, vegetables, and whole grains have been linked to high maternal income and education levels, and subsequently to good health in later stages of life [39,40]. While some studies reveal that parents and close relatives play an important role in early nutrition, the food industry is known for targeting mothers and encouraging the early introduction [41] of unhealthy food products and beverages that increase the risk of cardiovascular and metabolic diseases later in life [42].

As we noted above, the importance of nutrition for development in the first two years has been well-documented; however, there is little information on the impact of sociodemographic factors and the formation of DP in this period of life [43]. We now know that parents and children tend to share DPs [44], which is why establishing the sociodemographic factors that influence healthy or unhealthy DPs is more relevant than ever [45].

Identifying dietary patterns at an early stage would allow greater specificity in our recommendations and help us promote healthier complementary feeding DPs and other healthy lifestyles from the beginning of life [46]. Therefore, this review evaluates evidence of the association between sociodemographic factors and DP in children under 24 months.

## 2. Material and Methods

### 2.1. Types of Studies

We included observational studies (cohort) reported as full-text that were written in English and focused on the association between sociodemographic factors and DP in children under 24 months.

### 2.2. Types of Participants

The criteria of the studies included in this research focused on the following maternal sociodemographic factors: education, age, occupation, household income, marital status, ethnicity, parity, smoking during pregnancy, and BMI. We included studies carried out without time restrictions in countries with high, middle, and low incomes. We also included studies that used statistical dimensionality reduction techniques to identify DP, such as cluster analysis, factor analysis (principal component analysis), latent class analysis and reduced rank regression. Documents not available in English were excluded. Case-control studies, cross sectional, intervention studies, reviews, letters, and case reports were not considered.

The sociodemographic variables included in the review were categorized as follows. Education: none, primary or elementary, secondary, high school, or college and beyond. Occupation: unemployed, part-time employment, or full-time employment. Maternal age: <30 or ≥30 years. Number of family members: 1–4, 5–8 and 9 or more family members. Household income: percentage of regional poverty index or percentage poverty level. Parity: multiparous vs. primiparous. Marital status: married or unmarried. Ethnicity: white non-Hispanic, African American, Hispanic, Asian/Pacific Islander, and others. BMI: normal (18.5–24.9 kg/m^2^), overweight (25.0–29.9 kg/m^2^), obesity (30–39.9 kg/m^2^). Smoking status: never smoked, past smoker, and current smoker.

### 2.3. Data Collection and Analysis

#### 2.3.1. Search Strategy

**Figure 1:**
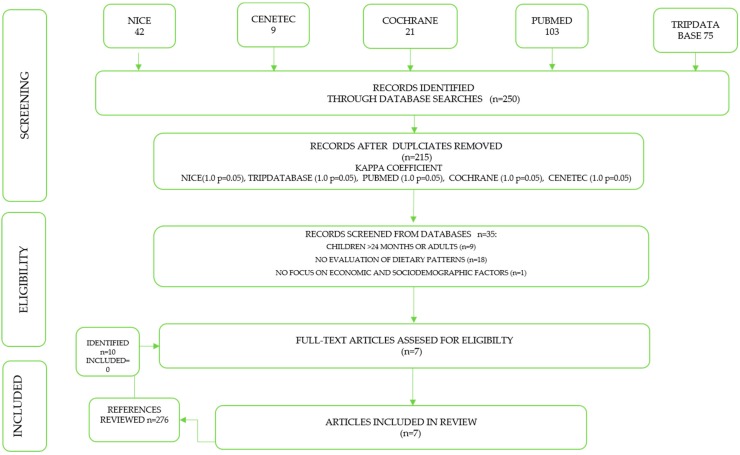
Literature review and selection criteria flow chart.

Individual strategies were used to research the databases MEDLINE (PubMed), Cochrane Library, NICE guidelines, TRIP database, and CENETEC. A hand searching was made into the reference of the included studies. The final search was updated in April 2019. The articles were selected for full-text reading and were screened individually for potentially relevant studies that may have been missed. The database search strategy is shown in Figure 1. As a way of including infants under 24 in our Pub Med database search, we used an age filter from 0 to 23 months and discarded search results with children older than 24 months.

The strategic search was completed under the PICOS framework, which stands for: Population = children under 24 months; Intervention = sociodemographic factors; Comparison = different DP; Outcome = DP; Study = observational. The Database search strategy included different word combinations for each database and references were managed with Endnote^®^ Web software v. 3.1.1. (Thomson ResearchSoft, California, United States) Duplicated references were eliminated.

#### 2.3.2. Selection of Studies

Selected studies were assessed by two methodological and clinical researchers (G.-C.C. and M.-S.L.) who independently reviewed titles, abstracts, and selection criteria (the selection process was done in Excel). Duplicated articles and those that did not meet inclusion criteria were not considered. Full-text study reports/publications were retrieved. It was not necessary to seek contact with the authors since all the information available in the articles was included. Relevant full-text study reports/publications were retrieved (Figure 1). We also assessed the risk of bias in every included study. A third reviewer (D.-G.E.) settled discrepancies.

#### 2.3.3. Data Extraction and Management

Extracted information included country, study design, study duration, follow-up (cohort studies), study location and participants; age range, dietary assessment method, sample size, DP derivation method, economic indicators (household income), identified DP, direction of association between DP and interventions; and sociodemographic factors.

In order to reduce the number of DP described in our selected studies, we identified two main DPs: “Healthy/Prudent” and “Unhealthy/Western”. The Healthy/Prudent DP was described as “adherence to guideline recommendations” according to age [1,46], and included fruits, vegetables, cereals, meat, eggs, olive oil, and whole grains. The “Traditional/Basic” DP was included in this group because it contained mainly local and home-cooked food which are commonly considered “healthy”. The Unhealthy/Western DP included processed energy-dense food, low fiber “fast food”, cookies, sweetened food, junk food, and puddings. For the present analysis, those DP including only beverages were not considered. The direction of association between sociodemographic factors and DP was described as: positive, converse or negative, exclusively when the associations were reported as statistically significant (*P* < 0.05) or if the 95% CI did not include a coefficient with a zero value or an odds ratio of 1.

#### 2.3.4. Assessment of Potential Bias in Included Studies

The methodologies of the selected studies and potential bias in each study were evaluated using the Strengthening the Reporting of Observational Studies in Epidemiology (STROBE) method.

Two independent reviewers categorized the risk of bias as high, moderate, or low. Disagreements were resolved by a third reviewer. Agreement between reviewers was calculated using Cohen’s kappa coefficient.

#### 2.3.5. Data Synthesis

Due to the variety of methods used to measure some variables of interest and due to a shortage of information in the included studies we decided not to carry out a meta-analysis in the present study.

## 3. Results

### 3.1. Results of the Search and Study Selection

In total, 250 relevant articles were identified using electronic databases. During our screening phase, we reviewed abstracts and eliminated 215 articles that were either duplicated or had studies with descriptive, case-control designs, and cross-sectional designs. Of the remaining 35 articles, 28 did not meet the PICOS criteria: of these; nine included children older than 24 months of age or adults, 18 were excluded because they did not analyze DP, while one study did not correlate economic and sociodemographic factors with DP (Figure 1). A concordance analysis assessed discrepancies between reviewers and revealed a “very strong” level of agreement. PubMed (0.55, *P* < 0.001), NICE (1.0, *P* < 0.001), Trip database (1.0, *P* < 0.001), Cochrane (1.0, *P* < 0.001), CENETEC (1.0, *P* < 0.001).

### 3.2. Included Studies

A total of seven studies based on “a posteriori” methods to derive dietary patterns were included in the final analysis. Of these, seven had cohort design. Updated searches were conducted in December 2018 and April 2019, and 10 new studies were identified. Of these, four articles met the phase one inclusion criteria but were eventually excluded due to their focus on maternal behavior (negative affectivity), or infant behavior (feeding difficulties), while others did not clearly describe DP associations with sociodemographic factors. No studies were included from this second review.

The sample size of studies included [47,48,49,50,51,52,53] ranged from 279 [47] to 9129 [48], and were carried out between 2012 and 2017. Of the seven studies included, three were carried out in the United States of America [47,50,53], two in Europe [48,51], one in Japan [52], and one in Australia [49]. In total, the analyzed studies included 7513 girls and 7587 boys with ages ranging from 6 to 24 months.

### 3.3. Dietary Patterns Analysis

The Food Frequency Questionnaire (FFQ) and multiple-pass 24-h dietary recall were the most common food intake (n = 3) evaluation instruments [47,49,50,51]. Three studies did not detail which instrument they used. The factor analysis method was the most frequently used method for deriving dietary patterns (n = 5) [48,49,51,52,53], while the other two studies used latent class analysis [47,50].

For the present review, we classified DP as “healthy/prudent” or “unhealthy/Western” pattern depending on which predominant foods in each study and in accordance with World Health Organization (WHO) international dietary recommendations for children’s age. Healthy patterns were considered as: “core food” [49], “meat, vegetables and dessert” [48], “herbs, fruit, and raw vegetables” [48], “breastfeeding or milk formula with fruit and vegetables” [47,50], “conscious health pattern or Mediterranean diet” [51], “fruit, vegetables, and high protein food” [52], and “solid food according to international guidelines for children’s age” [50] (Table 1).

On the other hand, “unhealthy/Western” DP were identified as follows: “no basic dietary pattern” [53], “mixed food pattern” [50], “unlimited dietary pattern”, “pre-made baby food pattern” [48], “milk formula and energy-dense foods” [47], “Western-like pattern” [51], “confectionaries and sweetened food pattern” [52], and “high sugar, fat, and protein food pattern” [49] (Table 1). Furthermore, Table 1 displays some DPs included as “healthy” and “unhealthy”, which were labelled as “basic combinations” [49].

### 3.4. Associations between Sociodemographic Factors and Dietary Patterns

Higher maternal age, ≥30 years old [47,48,49,52,53], and higher education levels were associated with “healthy” DPs [47,48,51,53]. Conversely younger mothers, <30 years old were positively associated with unhealthy DPs [48,49]. However, we found a positive association between high parental education levels and “unhealthy” DPs in one study. In general, low household income was associated with “unhealthy” DPs [53], however, this association was absent from one of the studies included [50]. Having more than one child and unemployment were positively associated with “unhealthy” DPs [48,49,50].

Three of the included studies [48,50,52] positively associated multiparity with “unhealthy” DP too, while one also found the same association with having fewer children [48]. Maternal BMI ≥ 30 kg/m^2^ during pregnancy was frequently associated with “unhealthy” DPs [48,51]. Other factors such as being married or unmarried were associated in converse directions with “healthy patterns” [48] and “unhealthy” patterns, respectively [47]. Being employed or unemployed carried the same association (Table 2) [52,53].

### 3.5. Risk of Bias in the Studies Included

Each article included was critically evaluated under the scope of STROBE criteria. Articles were classified as “high risk”, “moderate risk”, “low risk or unclear risk” and “very low risk” according to GRADE nomenclature (Table 3).

## 4. Discussion

This review suggests the presence of an association between sociodemographic factors (such as maternal education level, household income, maternal BMI, and maternal age) and DP in children under 24 months.

Five studies included in the present review showed that higher maternal education levels were associated with “healthy” DPs [47,48,49,52,53], whereas lower maternal education were associated with “unhealthy” DPs [48,51,52,53]. Our results are similar to previous studies where maternal education has been considered a determining factor for DP in mothers as much as in children [54,55,56,57]. For example, a study conducted by Hidaka et al. [58] on 190 American mother–child dyads with children between 2 to 4.5 years old evaluated and correlated DP with maternal SES factors such as education and household income. They found that mothers with healthier diets had on average higher SES scores (difference 1.05, CI 95%: 0.80 to 1.30) than mothers with “unhealthy” DPs. Furthermore, this study concluded that education was directly linked to favorable nutrient intake which was strongly associated with healthier nutritional behaviors in participating mothers. Financial support is another important factor frequently associated with DP in children. A systematic review conducted by Bazzano et al. [59] showed available qualitative research on DPs and practices in infants and young children from the perspective of parents and families in low-income settings in the USA. This review reported four categories of barriers obstructing recommended breastfeeding practices such as lack of support for breastfeeding from families, health workers and due to time poverty, and three categories of barriers to recommended complementary feeding practices showing how higher quality foods were replaced with lower quality foods for financial reasons, lack of diversity in available foods, lack of water for cooking, and inability to buy food. These last findings highlight the importance of economic factors when defining early DPs, especially those considered a risk factor for nutritional outcomes such as obesity, [60,61] malnutrition, and double burden malnutrition. However, further studies are required to corroborate these associations.

In this review we found an association between higher household income and healthy dietary patterns; however, all studies included in this review were carried out in developed countries and could not be extrapolated to populations with different characteristics. However, a previous systematic review developed in children and adolescents from countries with high, middle, and low incomes also reported an association between higher economic income and “healthy” DP [62].

Likewise, the results from this systematic review suggest that maternal age is associated with children’s DP. We found that children whose mothers were ≥30 years old had healthier DPs [48] mainly represented by high intake of fruits, vegetables, whole grains, seeds, cheese, and eggs. In contrast, children whose mothers were <30 years had unhealthier DPs characterized by sugar sweetened beverages, high-fat, highly processed foods, and candies [49]. It is possible that younger mothers may know less about nutrition than older mothers, however, further studies would be required before drawing any conclusions. An example of the importance of maternal age can be found in a cohort study performed on women in the United Kingdom, where women under 19 often consume unhealthy foods such as ultra-processed (energy-dense food) paired with a low intake of fruit and vegetables, while women aged 35 and older showed high consumption of fruits, vegetables, whole grains and starch [63]. Additionally, a study conducted in New Zealand [28] also found a positive association between increasing maternal age and “health conscious” and “fusion/protein” DPs, while also reporting the opposite “junk” and “traditional/white bread” pattern associations in younger mothers.

This review also found an association between maternal BMI ≥ 30.0 kg/m^2^ and unhealthy DPs in children [47,53]. Some studies have described an association between overweight or obese mothers and increased obesity risk in their children [64]. However, current literature on the association between the quality of parents’ diets and children’s diets is limited. There is little research on the relationship between parental and child DPs, particularly empirically derived DPs. Despite being limited, there is evidence in current literature supporting an association between the quality of parents’ diet and children’s diet, although theoretically derived patterns based on national nutrition guidelines differ between countries. Consequently, any significant relationships found using a particular country-specific index may not apply to other populations.

While this review found three studies that included an association between having more children and unhealthy dietary patterns [47,49,51] we did not find enough evidence to establish number of children in a family as a factor associated with DP. However, having more than two children may contribute to a less restrictive feeding environment due to elevated maternal stress levels, which may lead to unhealthy diets [65]. Mothers are influential in their children’s eating behaviors [66] and time-restrictions as a result of having multiple children and pressuring children to eat may favor unhealthy feeding practices.

A strength of this review is that it looks at studies conducted in countries across North America, Europe, Asia, and Oceania. As a result, we were able to describe similarities in dietary patterns around the world, as well as associations between sociodemographic factors and DP in children under 24 months of age [2,46,67,68]. Another strength is that information from those studies was collected through tools such as multiple-pass 24-h recall and FFQs or designed by professional staff [69]. Dietary pattern derivation methods (PCA and latent class analysis) used in the included studies are also validated [70]. The PCA method used for factor analysis added specific food items or food groups on the basis of how food items in the dataset correlated with each other [14].

Albeit, this review has limitations. The studies included in this review were carried out in developed countries and their results are unlikely to apply to developing or underdeveloped populations. Also, measurements such as household income were based on a regional index which makes them difficult to interpret.

## 5. Conclusions

The results of our systematic review suggest an association between sociodemographic factors (such as maternal education, maternal age, and household income) and dietary patterns in children under 24 months of age.

We recommend studies in developing and less developed countries to find out which sociodemographic factors are associated with dietary patterns in childhood in different contexts, as well as with different health outcomes such as malnutrition, obesity, and double burden malnutrition. Most of these are preventable with adequate complementary feeding. Meanwhile, we recommend the ongoing promotion of healthy eating habits that include fruits, vegetables, whole grains, seeds, cheese, eggs, and others, while reducing consumption of unhealthy sugary, high-fat, highly-processed foods and candies in the early stages of life [64].

## Figures and Tables

**Table 1:** Main characteristics of dietary patterns defined using an “a posteriori” approach.

Reference	Country	Sample Size	Age	Dietary Pattern Name and Food Contents
Bell LK et al., 2013 [49]	Australia	Infants 14 months: 552 Infants 24 months: 493	14 and 24 months	14 months Core foods: fruit, grains, no white bread, vegetables, cheese, eggs, nuts and seeds. Basic combination: white bread, milk and (non-core) spreads, juices, ice cream, food and beverages with no fruit or vegetables. 24 months Core foods: fruit, grains, non-white bread vegetables, cheese, eggs, walnuts, seeds, and water. Non-core food: (energy-dense, low-nutrient, sugars); sweetened beverages, spreads, high-fat fried/processed potatoes, snacks, chocolate, processed meat.
Chelsea MR et al., 2016 [50]	United States	Infants 1071	Infants 9 months	Breastfeeding (BF) Breastfeeding, fruits and vegetables (BFFV). Characterized by high probability of breastfeeding, fruits and vegetables, and low probability of high energy density. Breastfeeding low variety (BFLV). Represented by high probability of breastfeeding, low probability of fruits and vegetables and low variety. Formula Feeding (FF) Milk formula feeding, fruits and vegetables (FFFV). Characterized by high probability of fruits and vegetables, and low probability of energy-dense food. Milk formula feeding low variety (FFLV). Represented by high probability of formula, low probability of fruit and vegetable, and low food variety. Mixed: FF, low in fruits and vegetables and high probability of energy-dense food.
Smithers LG et al., 2012 [48]	England	Infants 6 months: 5129 Infants of 15 months: 3998	Infants 6 and 15 months	6 months Meat, vegetables, and desserts (vegetables cooked at home, potatoes, meat, fish, fruit or milk pudding, eggs). Biscuits, sweets, crisps: biscuits, chocolate, tea, sweets, bread, cola, fizzy drinks. Pre-made baby foods (commercial products, canned/jarred meat, processed meat), fruit puddings, vegetables or milk pudding, fish. Breastfeeding: breastfeeding, raw fruits and vegetables. 15 months Herbs, raw fruit and vegetables: legumes, raw vegetables, fruit, cheese, spices. Biscuits, sweets and crisps: fizzy drinks, sweets, crisps, biscuits, ketchup. Pre-made baby foods: rice cereal, rusks, baby meat, baby vegetables, baby milk and fruit pudding. Dorsal meat, vegetables and desserts. high consumption of meat products, home-cooked potatoes, milk puddings and vegetables.
Hohman EE et al., 2017 [47]	United States	279	Children 9 months	(BFV) Breastfeeding, fruits and vegetables. (BLV) Breastfeeding, low variety. (FFV) Milk formula, fruits and vegetables. (FLV) Milk formula, low variety. (FHED) Energy-dense food, juice, milk formula, sweetened drinks, French fries and sweetened food.
Kiefte de JJC et al., 2012 [51]	The Netherlands	2420	Children 14 months	Health conscious: fruits, vegetables, legumes, fish. Western-like: savory snacks, animal fats, confectionery, sweetened drinks.
Okubo H. et al., 2012 [52]	Japan	758	Infants from 16 to 24 months	Fruits, vegetables and high-protein foods: basic food, meat, fish, eggs, vegetables, fruits, yoghurt, green tea, Oolong tea. Confectionaries and sweetened beverages: fresh fruit juice, sweetened fruit juice, puddings, jellies, chocolate, rice crackers.
Xiaozhong W. et al., 2014 [53]	United States	1378	Infants from 6 to 12 months	High, sugar, fat, and protein pattern: sweetened drinks, sweetened food, French fries, fish/shellfish, nut products, eggs. Infant guideline solids: baby cereals, fruit, vegetables, meat/chicken. Formula. High dairy/regular cereal: cow’s milk, dairy products, fruit and vegetable juice, non-baby cereals/starches.

**Table 2:** Socioeconomic factors and their associations with dietary patterns defined in an “a posteriori” approach in children under 24 months.

Reference	Country	Study Location	Sample Size	Age Range	Diet Assessment Method/Dietary Pattern Method	Dietary Pattern Identified	Economic and Sociodemographic Factors Assessed	Association of Economic and Sociodemographic Factors and Dietary Patterns.
Bell LK et al., 2013 [49]	Australia	Brisbane, Adelaide, South of Australia	Infants 14 months: 552 Infants 24 months: 493	14 and 24 months	Multiple-pass 24-h recall/Principal Component Analysis (PCA)-factor analysis (varimax rotation).	14 months: “core food”, “basic combination” 24 months: “core food”, “non-core food”	Maternal: education level, age (during delivery), smoking during pregnancy, breastfeeding duration, marital status, weight, parity, economic status (decile), Australian nationality.	14 months Higher education levels (university) were associated with high consumption of “core food”. Younger mothers at time of delivery and smokers were conversely associated with the “basic combination pattern”. 24 months Australian nationality and younger mothers were associated with “non-core food”.
Chelsea MR et al., 2016 [50]	Unites States	Atlanta, Georgia	1071	9 months	Food Frequency Questionnaire (FFQ)/Class latent analysis	“Breastfeeding, fruit and vegetables” (BFFV), “Breastfeeding low variety” (BFLV) “Milk formula feeding, fruits and vegetables” (FFFV), “Milk formula feeding low variety” (FFLV) Mixed: “high energetic density”	Maternal: parity, age, excessive weight-gain, body mass index before pregnancy, ethnic group, education level, household income, postpartum depression, marital status.	Hispanic vs. non-Hispanic white race/ethnicity mothers were associated with “fruits and vegetables “and “formula feeding and low variety”. Multiparity was associated with “formula feeding and low variety patterns”. High school education or less was associated with both “formula feeding and low variety” and “mixed patterns”. Low household income was associated with the “formula feeding and fruit and vegetables pattern”.
Smithers LG et al., 2012 [48]	England	Avon (South-West of England)	Infants 6 months: 5129 Infants 15 months: 3998	Infants 6 and 15 months	Questionnaire not validated/PCA-factor analysis	6 months: 1. ”Meat, vegetables, desserts”; 2. “Biscuits, sweets, crisps”; 3. “Pre-made baby food”; 4. “Breastfeeding”. 15 months: 1. “Herbs, raw fruit and vegetables”; 2. “Biscuits, sweets and crisps”; 3. “Pre-made baby food”; 4. “Dorsal meat, vegetables and desserts”.	Maternal: education, age, social class, smoking, number of children, marital status, and body mass index.	6 months Infants from mothers with higher education levels were positively associated with dietary pattern 1. Younger mothers, with lower levels of education who smoked during pregnancy had higher body mass index and a larger number of children were positively associated with dietary pattern 2. Younger mothers, lower education and smoking during pregnancy were positively associated with dietary pattern 3. At 15 months Older mothers with higher education levels were positively associated with dietary pattern 1, however, older, unmarried mothers who only had one child scored highly for dietary pattern 3. Maternal body mass index ≥ 30 kg/m^2^ was associated with high dietary pattern 2 scores. Being married was associated with low pattern 4 scores.
Hohman EE et al., 2017 [47]	United States	Pennsylvania	279	9 months	FFQ/class latent analysis	“Breastfeeding, fruit and vegetables (BFV)” “Breastfeeding, low variety (BLV)” “Milk formula, fruits and vegetables (FFV)” “Milk formula, low variety (FLV)” “Milk formula, high energetic density food” (FHED).	Mother’s education level, age, household income, marital status, gestational weight gain, prenatal body mass index, return to work after 3 months.	Older, high-income, married, and high education-level mothers were less associated with milk formula, low variety and milk formula, high energetic density food. High pre-pregnancy body mass index was associated with milk formula, low variety and milk formula, and high energetic density food. Milk formula, low variety was associated with mothers who returned to work at 3 months. No differences were found between weight gain during pregnancy, child sex, body weight at birth (*Z* score), and use of childcare.
Kiefte de JJC et al., 2012 [51]	Holland	Rotterdam	2420	Children 14 months	FFQ/PCA-factor analysis (varimax rotation).	“Health conscious”, “Western-like”	Mother’s education, household income, marital status, smoking during pregnancy, alcoholism during pregnancy, prenatal body mass index, energy intake before pregnancy, number of children, arterial hypertension, hypercholesterolemia, paternal education, paternal body mass index, paternal diabetes, arterial hypertension, paternal age, smoking.	Low paternal education, low household income, parental smoking, high maternal body mass index during pregnancy, high intake of carbohydrates, and multiparity were associated with the “Western-like pattern”. High fiber intake during pregnancy and older parents were conversely associated with the “Western-like pattern”. Folic acid intake during pregnancy, high fiber maternal diet, and single parenthood were positively associated with the “healthy pattern”. Mothers who consumed alcohol during pregnancy and had a history of comorbidity, and those with daughters, were less associated to the “health conscious” pattern.
Okubo H. et al., 2012 [52]	Japan	Neyagawa Osaka	758	Infants from 16 to 24 months	Self-administered questionnaire/PCA-factor analysis (varimax rotation). Cluster analysis.	“Fruit, vegetables and high-protein foods” “Confectionaries and sweetened beverages”	Maternal age, pre-pregnancy body mass index, education (years), employment status, household income, family structure, married (yes/no), number of infants’ older siblings, cigarette smoking during pregnancy, physical activity, maternal dietary pattern.	Unemployed mothers, daily smokers during pregnancy, lower education levels, lower household income, higher number of children, were associated with “confectionary and sweetened beverages”. Non-smokers during pregnancy, high education levels, longer duration of breastfeeding, full-time employment, and higher household income with a rice, fish and vegetable intake pattern were less associated with “confectionary and sweetened beverages”. Mothers with more than 13 years of education and a rice, fish, and vegetable dietary pattern, were less associated with “confectionary and sweetened beverages”.
Xiaozhong W. et al., 2014 [53]	United States	Buffalo, New York	1378 1275	Infants from 6 to 12 months	Surveys/PCA-factor analysis (varimax rotation).	“High sugar, fat and protein pattern”, “infant guideline solids”, “formula milk”, “high dairy and regular cereal”.	Maternal: age, ethnicity, education level, married (yes/no), employment (yes/no), parity, gestational diabetes, gestational weight gain, pre-gestational body mass index, household income.	Low household income, maternal non-Hispanic Afro-American ethnicity, low education level, high body mass index were associated with the “high sugar, fat and protein dietary pattern. High household income, maternal non-Hispanic, white ethnicity, and high education were associated with “Infant guideline solids”. Emergency cesarean-section, higher maternal age, Asian-pacific ethnicity, and low gestational weight gain were associated with the “formula, baby cereal dietary pattern”. Vaginal non-induced, maternal non-Hispanic, white race/ethnicity, low education, being employed, and obesity were associated with “high dairy and regular cereal dietary pattern”.

**Table 3:** Data extraction of studies included in review (title study details and assessment of methodological limitations).

Author, Year, and Country of Study	Title	Source Type	Objective	Study Design, Analysis Method (AM)	Setting and Sample Size	Assessment of Methodological Limitations of Study (STROBE)/Quality of the Evidence using the Nomenclature of the GRADE Manual Valued at: High ⨁⨁⨁⨁; Moderate ⨁⨁⨁ ◯; Low ⨁⨁ ◯◯; and Very Low ⨁ ◯◯◯.
Bell LK et al., 2013, Australia [49]	Dietary patterns of Australian children aged 14 and 24 months, and associations with socio-demographic factors and adiposity	Journal: *European Journal of Clinical Nutrition*	To describe dietary patterns of Australian children aged 14 and 24 months; identify socio-demographic factors behind dietary patterns; examine associations between dietary patterns and child adiposity.	Secondary analysis, longitudinal study. Dietary patterns were extracted using Principal component analysis (PCA).	Purposive sampling, subjects were recruited in a two-stage process; mothers delivering healthy infants (37-week gestation, 2500 g) were approached for permission to be re-contacted approximately 3 months later for full enrolment in the study, 1 045 subjects.	No explanation regarding sample size calculation, interactions, missing data treatment. No information on loss of participants, and no flow chart/diagram shown. Moderate ⨁⨁⨁ ◯
Chelsea MR et al., 2016, United States, [50]	Patterns of Early Dietary Exposures Have Implications for Maternal and Child Weight Outcomes	Journal: *Obesity*	To identify distinct classes of infant dietary patterns at 9 months using latent class analysis; identify maternal and infant characteristics associated with infant dietary patterns; test whether infant dietary class membership is associated with child and maternal weight.	Cohort study, latent class analysis was used to identify discrete, mutually exclusive latent classes, based on 9-month Food Frequency Questionnaire (FFQ) data.	Convenience sampling, women were recruited late in pregnancy to participate in the IFPS II project, conducted in 2005. All data were self-reported by mothers on mailed surveys, 1 807 continued participation through 1 year.	No explanation of possible bias in sources, interactions, and missing data treatment. No information on sampling methods, on loss of participants, and no flow chart/diagram or information on approval from the ethics committee shown. Moderate ⨁⨁⨁ ◯
Smithers LG et al., 2012, England, [48]	Associations between dietary patterns at 6 and 15 months of age and sociodemographic factors	Journal: *Journal of Clinical Nutrition*	To describe dietary patterns in early life and their associations with maternal and infant sociodemographic characteristics.	Cohort study, PCA was used to explore latent diet patterns on a continuous scale.	Convenience sampling, all pregnant women residing in Avon, southwest England, were invited to participate. The core ALSPAC sample consists of 14,541 pregnancies with 13,988 infants alive at 1 year.	No explanation regarding sample size calculation, interactions, missing data treatment. No information on loss of participants. Moderate ⨁⨁⨁ ◯
Hohman EE et al., 2017, United States, [47]	INSIGHT Responsive Parenting Intervention is Associated with Healthier Patterns of Dietary Exposures in Infants	Journal: *Pediatric Obesity*	INSIGHT study: Latent class analysis (LCA) approach to identify patterns of milk and complementary feeding in 9-month-old infants., Explored the relationship between the BMI and the effect of maternal and infant characteristics on dietary pattern class membership, determine whether dietary pattern class membership differed between RP and control groups.	Cohort study. LCA was used and based on the infant FFQ data.	Primiparous mothers-newborns dyads (n = 291) were randomized to the intervention INSIGHT, RP or control. Latent class analysis identified patterns of dietary exposure at 9 months (cohort).	No explanation of interactions, missing data treatment. No explanation of source bias. Moderate ⨁⨁⨁ ◯
Kiefte de JJC et al., 2012, Holland, [51]	Socio-demographic and lifestyle determinants of ‘Western-like’ and ‘Health conscious’ dietary patterns in toddlers	Journal: *British Journal of Nutrition*	To identify common dietary pattern in toddlers and to explore parental and child indicators of these dietary patterns.	Cohort prospective study, principal component analysis and varimax method by maximizing the sum of the variance of the loading components was used.	Convenience sampling the study was embedded in a population-based prospective cohort study in Rotterdam, the Netherlands. In total, 9778 mothers with a delivery date between April 2002 and January 2006 were enrolled but only 3643 (72%) were eligible for analysis.	No explanation of interactions, missing data treatment, loss of participants or confusion factors. No explanation of source bias. Moderate ⨁⨁⨁ ◯
Okubo H. et al., 2012, Japan, [52]	Dietary patterns in infancy and their associations with maternal socio-economic and lifestyle factors among 758 Japanese mother–child pairs: the Osaka Maternal and Child Health Study	Journal: *Maternal and Child Nutrition*	To identify dietary patterns in US infants at ages of 6 and 12 months, sociodemographic differences in these patterns, and their associations with infant growth from ages of 6 to 12 months.	Prospective cohort study. First, they adopted an a posteriori approach, using principal component analysis. Second, they conducted several runs with the number of clusters varied from two to six.	Purposive sampling all pregnant women in the Osaka Prefecture, were recruited between 2001 and 2003. Of 3639 eligible women, 627 (17.2%) agreed to participate in the survey. An additional 375 pregnant women living in other municipalities were also enrolled between 2001 and 2003. The final analysis consisted of 758 mother–child pairs.	No explanation about statistical methods used to control confusion factors, subjects were not randomly sampled, no explanation about missing data treatment, and they assumed high bias risks sources, and lack of precision on some study variables (socio-economic status). Very low ⨁ ◯◯◯.
Xiaozhong W. et al., 2014, United States, [53]	Sociodemographic Differences and Infant Dietary Patterns	Journal: *Pediatrics*	To identify dietary patterns among infants aged 16–24 months, and the influence of maternal socio-economic and lifestyle characteristics on identified dietary patterns.	Secondary analysis—longitudinal study. Principal component analysis and Orthogonal transformation (varimax) to rotate the original derived components were used.	Purposive sampling, this longitudinal study followed pregnant women from late pregnancy through their infant’s first year of life. The original study sample consisted of 4902 pregnant women and 3033 full-term newborns. The final growth analysis only included the 530 infants who had complete data.	No explanation of statistical methods used to control confusion factors. No explanation of missing data treatment. Moderate ⨁⨁⨁ ◯
